# Disulfidptosis and androgenic cancers: from molecular mechanisms to clinical applications and future translational research

**DOI:** 10.3389/fendo.2026.1783929

**Published:** 2026-02-25

**Authors:** Xueyang Wang, Juan Zhao, Qiming Li, Jiaqing Chang, Weiwei Zhao, Xiping Xing

**Affiliations:** 1 Gansu University of Chinese Medicine, Lanzhou, China; 2 Affiliated Hospital of Gansu University of Chinese Medicine, Lanzhou, China

**Keywords:** clinical applications, disulfidptosis, future transformation, male reproductive cancers, molecular mechanisms

## Abstract

Disulfidptosis, a newly discovered form of programmed cell death, has garnered significant attention in tumor biology and reproductive system research in recent years, particularly demonstrating importance in urological cancer studies. Prostate cancer, testicular cancer, and bladder cancer are highly prevalent malignant tumors among men globally. Modern medical research reveals their complex pathogenesis and the limited efficacy of traditional treatments, necessitating the identification of novel therapeutic targets. Disulfidptosis influences tumor cell survival and death by regulating the formation and cleavage of intracellular protein disulfide bonds, highlighting its pivotal role in tumorigenesis and progression. This paper systematically reviews the molecular mechanisms of disulfidptosis, elucidates its regulatory role in male cancer cells—including key regulatory genes and therapeutic target potential—and discusses its application value and challenges as a potential therapeutic target based on clinical research. It aims to deepen understanding of disulfidptosis regulation, provide new insights and strategies for future precision treatment and clinical translation of male cancers, and drive innovation in related therapeutic approaches.

## 1 Introduction

Male reproductive cancers pose a significant threat to men’s health, with both incidence and mortality rates showing an upward trend. Its incidence rate has reversed from a downward trend of 6.4% per year from 2007 to 2014 to an increase of 3.0% per year from 2014 to 2021. Additionally, over the past decade, the incidence rate among men under 55 has increased by 2.6% per year, among men aged 55–69 by 6.0% per year, and among men aged 70 and older by 6.2% per year (1). Moreover, data indicates that between 2005 and 2020, the incidence of prostate cancer among Iranian men rose from 11.46 to 25.67 cases per 100,000, while testicular cancer increased from 2.39 to 5.05 cases per 100,000 (2). A similar Scottish study on individuals with intellectual disabilities found higher incidence and mortality rates of reproductive system tumors in this population compared to the general population, suggesting special needs for cancer prevention and treatment in this group (3). Additionally, men with inflammatory bowel disease exhibit significantly increased risk of male reproductive system cancers, indicating that chronic inflammation may be associated with the development of urological cancers (4). Epidemiological data underscores the severity of urological cancers and the complexity of their multifactorial pathogenesis, making novel therapeutic approaches and strategies a research priority. In recent years, the diversity of tumor cell death mechanisms has provided new targets for cancer treatment. Currently, cell death pathways are primarily categorized into regulated cell death (RCD) and unregulated cell death. RCD encompasses multiple forms including apoptosis, necroptosis, pyroptosis, and autophagy-dependent cell death (5, 6). Among these, immunogenic cell death (ICD) has emerged as a key focus in cancer immunotherapy due to its ability to activate anti-tumor immune responses (7, 8). Additionally, novel cell death forms such as ferroptosis have been identified as closely linked to tumorigenesis and progression, emerging as new therapeutic targets (9, 10). This paper systematically reviews the molecular mechanisms of disulfidptosis, thoroughly analyzes its role in urological cancers and clinical translation potential, providing crucial theoretical foundations for precision treatment in urological malignancies.

## 2 Theoretical foundations of thioremediation

**Figure 1:**
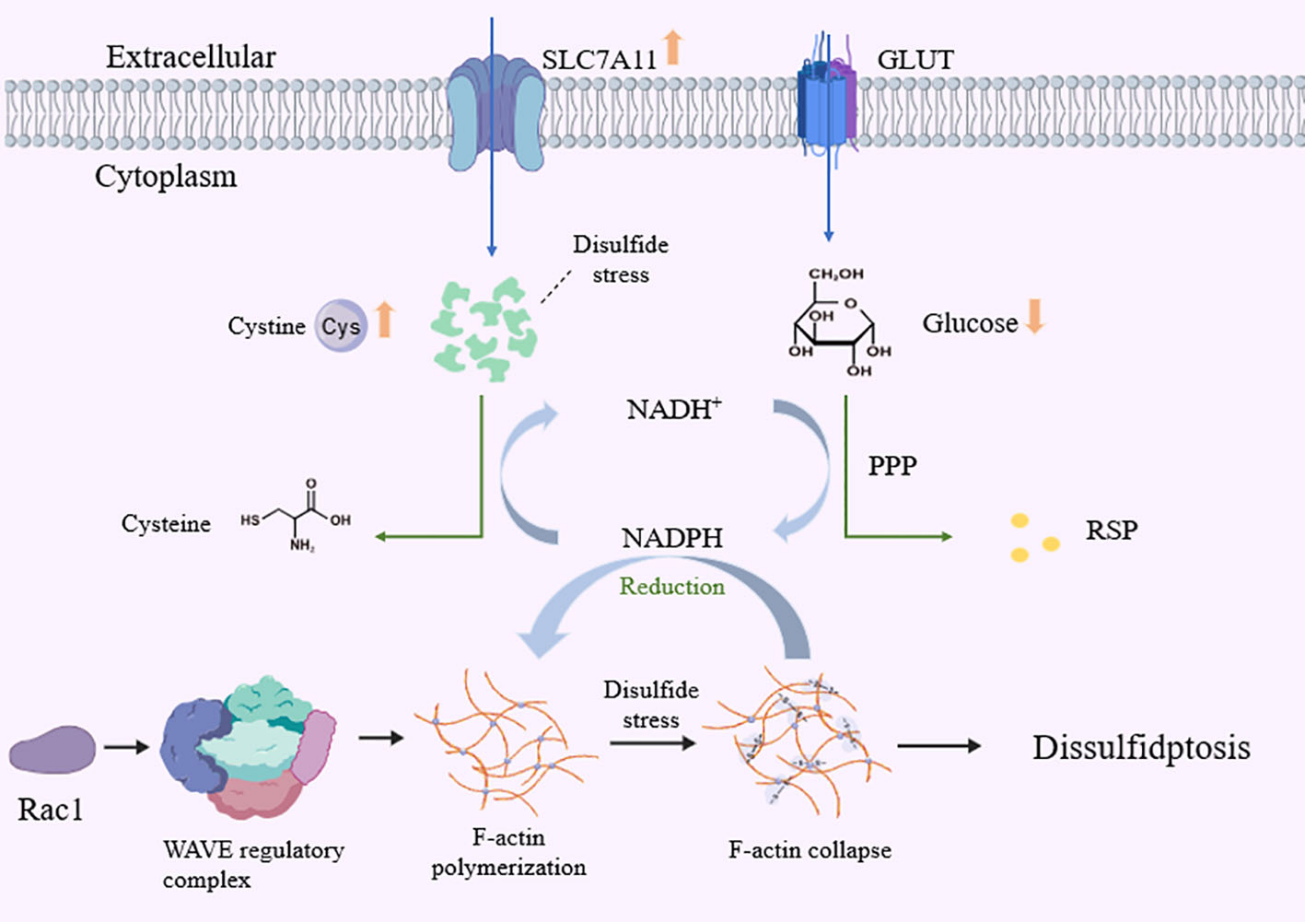
Molecular mechanism of disulfidptosis cell death.

Research has identified a novel form of cell death—disulfidptosis—whose unique mechanism is closely linked to the metabolic state of tumor cells. Disulfidptosis is triggered by the abnormal accumulation of intracellular disulfide bonds, particularly evident in tumor cells that overexpress the cystine transporter SLC7A11 and are subjected to glucose starvation (11, 12), This process involves cytoskeletal proteins, specifically the formation of abnormal intermolecular disulfide bonds in actin, the aggregation of F-actin filaments, and cytoskeletal collapse. This cascade ultimately leads to cell death (13, 14). Mechanistic studies indicate that the WAVE Regulatory Complex (WRC) and Rac protein also modulate this process. Inactivation of WRC suppresses disulfidptosis, while sustained activation of Rac promotes it (11) (**Figure 1**). Furthermore, Liu et al. demonstrated that glucose transport inhibitors induce disulfidptosis in tumors overexpressing SLC7A11, thereby inhibiting tumor growth. This suggests potential therapeutic value for this mechanism in cancer treatment (11). The specific mechanisms of disulfidptosis in urological cancers remain under investigation. However, existing studies indicate that abnormal SLC7A11 expression in various tumors is associated not only with cellular antioxidant defense but also with cell death pathways (15, 16). Furthermore, members of the protein disulfide isomerase (PDI) family, as key enzymes regulating disulfide bond formation and reduction, are closely associated with tumor cell survival and death. Research by Mary E et al. indicates that PDI inhibitors have been demonstrated to induce tumor cell death (17, 18). Research by Hou et al. also revealed that PDI catalyzes the dimerization of nitric oxide synthase (NOS), mediating the accumulation of intracellular reactive oxygen species (ROS) and lipid peroxides, thereby promoting ferroptosis (19, 20). In summary, regulating disulfide-related enzymes and associated metabolic pathways may offer novel therapeutic strategies for urological cancers.

Clinically, although the translational application of disulfide-mediated death is still in its infancy, related therapeutic strategies are beginning to take shape. Henderikus E et al. demonstrated that inducing metabolic stress in tumor cells via glucose metabolism-targeting drugs, combined with immunotherapy, enhances clinical efficacy (21, 22). Zeng et al. utilized nanotechnology to precisely regulate redox states within tumor cells, promoting immunogenic cell death and strengthening antitumor immunity (23, 24). Furthermore, in-depth studies on cell death mechanisms in male cancers such as prostate and testicular cancer have laid a foundation for precision medicine and personalized treatment (25, 26).

## 3 Molecular mechanisms of disulfidptosis

### 3.1 Disulfide bond formation and intracellular redox regulation

Disulfide bonds are covalent links formed between two cysteine residues in the tertiary structure of proteins, playing a crucial role in maintaining protein stability and function. Disulfide bond formation typically occurs in more oxidized regions of the cellular redox environment, such as the endoplasmic reticulum and mitochondrial intermembrane space, through the oxidation of the thiol group (–SH) of cysteine to form S–S bonds. Studies indicate that plasma proteins in blood, such as fibrinogen and α2-macroglobulin, exhibit multiple disulfide bond states. This suggests that the formation of protein disulfide bonds may be dynamic and influence protein function (27). Intracellular redox balance serves as a key regulator of disulfide bond dynamics. By modulating the redox state of cysteine residues, it influences disulfide bond formation and cleavage. In oxidized states, disulfide bond formation is promoted, enhancing protein stability; conversely, in reduced states, bonds readily cleave, inducing structural changes that regulate cellular functions. For instance, glutathione (GSH) and glutathione disulfide (GSSG) play vital roles in maintaining intracellular reducing conditions. Oxidation-induced protein disulfide bonds can be modified via glutathionylation, participating in signal transduction and cellular stress responses (28). PDI is a key enzyme regulating disulfide bond formation and rearrangement. PDI and its homologs catalyze protein disulfide bond isomerization and redox reactions, ensuring proper protein folding. Zhang et al. discovered that certain virus-encoded AC81 proteins exhibit PDI-like functions, participating in viral protein disulfide bond formation, indicating PDI’s crucial role in viral life cycles (29). Additionally, intracellular thiol oxidases contribute to the catalytic generation of disulfide bonds, maintaining the oxidative state of proteins (30). The activity of these enzymes depends on the cellular redox environment, forming a dynamic regulatory network for disulfide bonds. In summary, disulfide bonds serve as crucial components of protein structure and function. Their formation and cleavage undergo precise regulation by cellular redox states. Key enzymes such as protein disulfide isomerase ensure the correct formation and isomerization of disulfide bonds through catalytic action, thereby influencing cellular fate and function. This dynamic regulation provides a robust foundation for the molecular mechanisms of disulfidptosis.

### 3.2 Disulfidptosis related signaling pathways

Signaling pathways associated with disulfidptosis primarily revolve around oxidative stress responses, mitochondrial dysfunction, and disulfide modifications of relevant proteins. Xu et al. demonstrated that oxidative stress induced by copper-dithiocarbamate complexes in tumor cells promotes disulfide polymerization of associated proteins, leading to mitochondrial dysfunction and cell death (31). The specific mechanism involves triple amplification of oxidative stress, including GSH depletion, ROS production, and abnormal protein ubiquitination, leading to the synergistic action of apoptosis, ferroptosis, and disulfidptosis. Additionally, changes in calcium ion (Ca^2+^) influx during disulfidptosis have been found to be closely associated with cell death. Calcium signaling regulates multiple cellular functions, and abnormal Ca^2+^ levels can cause mitochondrial dysfunction and oxidative stress, thereby accelerating disulfidptosis (32). Recent research by Martyna et al. further revealed that mitochondrial voltage-dependent anion channels (VDACs), particularly VDAC3, sense intracellular redox states through their cysteine residues and participate in regulating mitochondrial metabolism and function (33). Changes in their oxidative state may serve as one of the signals triggering disulfidptosis. Furthermore, copper-dithiocarbamate complexes within signaling molecules can target the NPL4 protein, disrupting the ubiquitin-proteasome system. This induces protein toxicity stress, subsequently activating heat shock and unfolded protein responses, ultimately leading to cell death (34). Similar mechanisms have been observed in multiple copper-dithiocarbamate complexes, suggesting that NPL4 protein aggregation may be a key trigger for disulfidptosis. Additionally, regulatory factors of disulfidptosis include inhibition of the antioxidant enzyme GPX4 and reduced GSH levels, which exacerbate lipid peroxidation, promoting cell membrane rupture and death (35). Li et al. found that oxidative modification of protein disulfide bonds also participates in regulating cellular signaling and death processes (36). In summary, disulfidptosis is mediated through a series of interconnected signaling pathways, including oxidative stress-induced abnormal aggregation of protein disulfide bonds, calcium signaling regulation, mitochondrial dysfunction, abnormal protein ubiquitination, and lipid peroxidation. Among these, the copper-dithiocarbamate complex serves as a key inducer of disulfidptosis, activating this pathway through multiple mechanisms and offering novel therapeutic targets for diseases such as cancer.

### 3.3 Distinctions and connections between disulfidptosis and other forms of cell death

Disulfidptosis, as a newly discovered form of cell death, possesses unique molecular characteristics while also exhibiting connections to other forms of cell death (see **Table 1**). Compared to classical apoptosis, disulfidptosis does not display typical apoptotic morphological features such as nuclear condensation and chromatin marginalization. Instead, it relies more heavily on the abnormal formation of protein disulfide bonds and mitochondrial dysfunction (37). The distinction lies in apoptosis primarily mediating programmed cell death via caspases, whereas disulfidptosis involves protein disulfide rearrangement and copper ion-mediated oxidative stress. Compared to necrosis, disulfidptosis induces changes in cell membrane permeability through specific molecular events, independent of traditional inflammatory signaling pathways (38), which partially distinguishes it from the passive cell rupture and inflammatory response associated with necrosis. Pyroptosis is an inflammatory cell death that relies on gasdermin to form membrane pores, leading to cell rupture and the release of inflammatory mediators (39). Flux death shares similarities with pyroptosis in both involving changes in cell membrane permeability and being inducible by oxidative stress. However, flux death does not depend on gasdermin activation, with its mechanism focusing more on the formation and aggregation of protein disulfide bonds. Ferroptosis is an iron-dependent form of cell death driven by lipid peroxidation, primarily characterized by reduced mitochondrial fission and increased membrane density (40). Although both ferroptosis and ferroptosis involve oxidative stress and mitochondrial dysfunction, ferroptosis primarily depends on lipid peroxidation. However, recent studies indicate that ferroptosis may exhibit cross-regulation and synergistic effects with multiple cell death mechanisms such as apoptosis and pyroptosis. For instance, the copper-dithiocarbamate complex not only induces ferroptosis but also activates ferroptosis and apoptosis (31). This indicates that during cellular life processes, disulfidptosis functions as an independent and significant pathway that interacts with other forms of cell death to jointly maintain cellular homeostasis under both physiological and pathological conditions. In summary, disulfidptosis differs from traditional cell death modes such as apoptosis, necrosis, and pyroptosis in both molecular mechanisms and morphology. It exhibits unique characteristics of abnormal protein disulfide bond aggregation and mitochondrial dysfunction while maintaining complex connections and cross-regulation with other death forms, offering new insights into the diversity of cell death.

**Table 1:** Differences between disulfide bond cleavage and other forms of cell death.

Forms of cell death	Core molecular mechanism	Main morphological characteristics	Critical dependency	Association with Disulfidptosis
Disulfidptosis	Abnormal formation and polymerization of protein disulfide bonds and mitochondrial dysfunction (13)	No atypical nuclear enrichment/chromatin marginalization, altered cell membrane permeability	Copper-mediated oxidative stress	As a core reference, it can cross-regulate with various forms of cell death, serving as an independent programmed cell death pathway (15).
Apoptosis	caspase-mediated programmed cell death (38)	nuclear condensation, chromatin marginalization	caspases	Cross-regulation + Synergy: Copper-dithiocarbamate complexes can simultaneously induce disulfide death and activate the apoptosis pathway, representing programmed cell death mediated by distinct pathways with shared regulatory nodes (31).
Necrosis	Passive cell rupture activates conventional inflammatory signaling pathways (84)	Cell rupture, triggering an inflammatory response	No specific mediator molecules (passive process)	No significant synergy/antagonism: Both can induce changes in cell membrane permeability, but disulfide death is driven by specific molecular events and does not rely on inflammatory signaling pathways. The core triggering mechanisms show no significant overlap with regulatory pathways (15, 85).
pyrolysis	The gasdermin protein forms membrane pores, releasing inflammatory mediators (39).	Cell rupture and release of inflammatory mediators	gasdermin protein, oxidative stress	All can be induced by oxidative stress, and all exhibit changes in cell membrane permeability; there is no common node for gasdermin activation, only different death branches downstream of oxidative stress, with no direct antagonistic relationship (86, 87).
Ferroptosis	Lipid peroxidation-driven cell death (40)	reduced mitochondrial folds and increased membrane density	iron ions, oxidative stress, mitochondrial dysfunction	Cross-regulation + Synergy: Both involve oxidative stress and mitochondrial dysfunction. The copper-dithiocarbamate complex can simultaneously induce disulfide death and activate the ferroptosis pathway, representing a synergistic regulatory mechanism downstream of oxidative stress (31).

## 4 Role of disulfidptosis in urological cancers

### 4.1 Prostate cancer and disulfidptosis

Prostate cancer, one of the most common cancers in men, has seen increasing research focus on the expression and regulatory networks of disulfide-dependent death-associated molecules within its cells. Studies indicate that PDIA1 and PDIA5, members of the PDI family, are highly expressed in prostate cancer cells, with their expression regulated by the androgen receptor (AR) signaling axis. The stability and function of AR can influence cancer cell growth and survival. Xie et al. discovered that knocking out or pharmacologically inhibiting PDIA1/PDIA5 expression induces redox imbalance and mitochondrial dysfunction, suppresses cell proliferation, and induces cell death. Furthermore, this approach enhances the therapeutic efficacy of the AR antagonist enzalutamide, suggesting PDIA1/PDIA5 may represent potential therapeutic targets for prostate cancer (41). Transcriptomic data reveal that genes associated with disulfidptosis, such as UBASH3B, ANP32E, and PRC1, are upregulated in prostate cancer progression and closely correlated with high Gleason scores. These genes may serve as molecular biomarkers for disease staging and prognosis assessment. Furthermore, risk scoring models constructed based on these genes demonstrate robust prognostic predictive capabilities. Patients with different subtypes exhibit significant differences in immune infiltration, tumor mutational burden, and signaling pathway activity, suggesting that disulfidptosis related pathways participate in tumor microenvironment regulation and disease progression (42, 43), providing further insights for therapeutic strategies. Disulfidptosis also influences prostate cancer cell proliferation and migration by regulating cellular energy metabolism and glucose uptake. Kang et al. found that high SLC7A11 expression promotes cell migration, while its inhibitors—such as the glucose transporter inhibitor BAY-876—effectively suppress survival in SLC7A11-overexpressing cells, revealing the potential impact of metabolic pathway regulation on disulfidptosis (44). Furthermore, the targeted delivery system described by Yu et al., which carries a disulfide bond-forming agent—hyaluronic acid-thiobisulfite-geraniol (HA-SS-Geraniol) conjugate, can specifically target CD44-positive prostate cancer cells, inducing mitochondrial-mediated apoptosis and significantly inhibiting tumor growth. This further validates the therapeutic potential of the disulfidptosis mechanism in prostate cancer treatment (45). Various studies indicate that the expression of disulfidptosis related proteins is closely associated with treatment response in prostate cancer. Transcriptomic and immunohistochemical analyses of patient samples in clinical studies revealed that highly expressed disulfidptosis gene subtypes correlate with favorable survival prognosis and immune cell infiltration, whereas low-expression subtypes are associated with treatment resistance and disease progression (44, 46). Basic research has demonstrated that inhibiting S-S death-related molecules effectively delays tumor growth, suggesting their potential as therapeutic targets. Furthermore, studies employing nanomaterial-drug conjugate strategies to enhance S-S death induction have achieved considerable progress in prostate cancer. These approaches not only improve drug targeting and bioavailability but also mitigate systemic toxicity, exhibiting broad prospects for clinical translation (47). In summary, the mechanism of disulfidptosis in prostate cancer is complex, involving the expression regulation of multiple molecules and the interaction of signaling pathways. However, in-depth analysis of its regulatory networks and mechanisms not only aids in understanding the biological characteristics of prostate cancer but also provides more theoretical foundations and practical guidance for developing novel therapeutic strategies based on disulfidptosis.

### 4.2 Disulfidptosis in testicular and bladder cancers

In testicular cancer, especially seminoma tumors, high SLC7A11 protein expression and mRNA levels are often observed, which may be associated with the proliferation, survival, and initial response to chemotherapy (cisplatin) of certain subtypes, or their unique metabolic reprogramming and oxidative stress states (48). This high expression pattern suggests that cells of testicular germ cell tumors (GCTs) may be in a “preparatory state for disulfidptosis”, that is, high baseline cystine intake, which creates conditions for subsequent induction of disulfide, and testicular GCTs are abnormally sensitive to oxidative stress and metabolic interference compared with non-germ cell tumors, which also provides a theoretical basis for treatment using the disulfide pathway (49). The “push” strategy aims to further increase cystine influx and intracellular disulfide bond pressure, i.e., upregulate SLC7A11 expression, but this needs to be precisely controlled, as SLC7A11 overexpression itself may trigger death under oxidative stress conditions (50), and a more feasible “pull” strategy is to limit the reduction buffering capacity of cells, especially by inhibiting glucose transport or glycolysis key enzymes to limit NADPH production (51), which has been shown to be The combined use of endoplasmic reticulum stress inhibitors and glucose transporter inhibitors can significantly inhibit tumor growth by inducing disulfide bond stress and disulfidptosis (52).

Studies have shown that the expression of SLC7A11 differs significantly in different molecular subtypes and clinical stages (53), which means that not all bladder cancer cells have the same sensitivity to disulfiosis induction SLC7A11, and it is speculated that some high-grade, aggressive bladder cancers may be upregulated to cope with stronger oxidative stress and support their rapid growth and metastasis, but this metabolic dependence may also become its Achilles heel. making it more prone to disulfidptosis under certain conditions (50). In research exploration, drugs targeting this pathway are combined with existing therapies to overcome drug resistance, such as nanotechnology-driven strategies that have been shown to induce disulfidptosis without SLC7A11 dependence and effectively fight bladder tumors *in vivo* experiments, and even reverse cisplatin resistance (53).

In summary, although the research on disulfidptosis in testicular cancer and bladder cancer is in its infancy, the revelation of its molecular mechanism has attracted widespread attention.

### 4.3 Disulfidptosis and resistance mechanisms in androgenic cancers

In common treatments for male cancers such as chemotherapy and targeted therapy, drug resistance is a major obstacle affecting efficacy. The role of disulfidptosis mechanisms in the development of resistance should not be overlooked. In prostate cancer, overexpression of PDIA4—a member of the protein disulfide isomerase family—is closely associated with docetaxel (DTX) resistance. Research by Qian et al. demonstrates that PDIA4 suppresses apoptosis by activating the Akt signaling pathway, thereby promoting the survival of drug-resistant cells. Knocking down PDIA4 significantly enhances sensitivity to chemotherapy drugs, suggesting it as a potential target for reversing drug resistance (54). The expression levels of disulfidptosis related genes also correlate with tumor cell responsiveness to immune checkpoint inhibitors. For instance, subtypes with high disulfidptosis gene expression exhibit greater immune cell infiltration and sensitivity to immunotherapy, suggesting that modulating disulfidptosis may improve immune tolerance states and enhance treatment efficacy (43, 44). Experimental data from Kang et al. further demonstrate that modulating the disulfidptosis pathway—such as selectively targeting SLC7A11-overexpressing cells with glucose transporter inhibitors (BAY-876)—can reverse drug-resistant phenotypes (44). Clinically, current therapeutic strategies targeting disulfidptosis mechanisms include developing inhibitors against protein disulfide isomerase, utilizing nanocarriers to deliver disulfide-cleaving drugs, and employing multimodal approaches combining chemotherapy and immunotherapy to overcome resistance to both chemotherapy and targeted therapies. Previous basic research has also demonstrated that these strategies can effectively inhibit tumor growth, prolong survival, and exhibit relatively controllable side effects, indicating promising potential for clinical translation (45, 47). In summary, disulfidptosis not only contributes to the initiation and progression of urological cancers but also profoundly influences therapeutic resistance mechanisms. Future targeted therapies based on disulfidptosis may represent a significant breakthrough in overcoming drug resistance in urological cancers, offering patients more effective personalized treatment options.

## 5 Clinical applications and future prospects of disulfidptosis

### 5.1 Potential advantages over traditional biomarkers

Biomarkers related to disulfidptosis, particularly SLC7A11, demonstrate unique advantages over traditional prostate-specific antigen (PSA) in the diagnosis of male urological cancers. As the cornerstone for prostate cancer screening and monitoring, the limitations of PSA are becoming increasingly evident. PSA levels can elevate in various conditions, including prostate cancer, benign prostatic hyperplasia (BPH), and prostatitis, leading to a high false-positive rate and consequently unnecessary invasive examinations and overtreatment (55). Case reports have shown that patients with extremely high PSA levels (exceeding 3000 ng/mL) may still only have localized prostate cancer rather than necessarily metastatic disease, highlighting the inadequacy of PSA in distinguishing disease aggressiveness (56). In contrast, the upregulation of disulfidptosis biomarkers is directly associated with specific metabolic stress and malignant phenotypes of tumor cells. As a cystine transporter, high expression of SLC7A11 is a key mechanism by which cancer cells respond to oxidative stress, resist ferroptosis, and is closely linked to enhanced tumor aggressiveness (49). Therefore, detecting biomarkers such as SLC7A11 can not only indicate the presence of cancer but also reflect the intrinsic, more aggressive biological characteristics of the tumor, potentially enabling more specific differentiation between malignant and benign lesions and providing a more precise basis for clinical decision-making.

The value of disulfidptosis biomarkers lies not only in their diagnostic specificity but also in their ability to dynamically reflect tumor biological behavior. High expression of SLC7A11 is not merely a static marker but is directly linked to malignant phenotypes such as oxidative stress resistance and ferroptosis evasion (15). This association implies that by detecting these biomarkers, clinicians can not only diagnose the presence of cancer but also preliminarily assess the invasive potential of the tumor, thereby facilitating more accurate risk stratification. Studies have indicated that SLC7A11 expression is further upregulated in castration-resistant prostate cancer (CRPC) (44). The diagnosis and therapeutic monitoring of CRPC represent significant clinical challenges, as changes in traditional PSA during this stage may be influenced by various therapeutic interventions, leading to reduced specificity. In contrast, changes in SLC7A11 expression may more directly reflect the metabolic reprogramming and survival stress during the transition to CRPC. Thus, it holds potential as a novel dynamic biomarker for monitoring disease progression to CRPC or assessing treatment response in CRPC, offering new insights to overcome bottlenecks in the clinical management of CRPC.

### 5.2 Potential in early diagnosis and monitoring of minimal residual disease

Metabolic alterations associated with disulfidptosis may occur during the early stages of tumorigenesis, providing a theoretical basis for their application in early diagnosis. To meet the demands of rapid proliferation, tumor cells undergo significant metabolic reprogramming, including enhanced reliance on cystine uptake and redox balance (57). Such metabolic changes may precede the appearance of radiologically detectable masses. Theoretically, using highly sensitive metabolic imaging techniques or liquid biopsy methods to detect relevant metabolic changes in the tumor microenvironment or circulatory system could potentially indicate the presence of tumors before they are detectable by traditional imaging methods. For example, molecular imaging techniques such as prostate-specific membrane antigen (PSMA)-based PET/CT have demonstrated exceptional capabilities in detecting prostate cancer recurrence at very low PSA levels (58). Disulfidptosis biomarkers also hold significant potential in monitoring minimal residual disease (MRD) or early recurrence after treatment. Currently, PSA is the primary indicator for monitoring prostate cancer recurrence post-treatment, but its interpretation at very low levels is challenging, and PSA elevation may lag behind actual disease progression (59). For patients with atypical PSA responses or those at risk of recurrence despite extremely low PSA levels, monitoring the levels of SLC7A11 or its related metabolites carried by tumor-derived exosomes in the blood may provide earlier and more specific signals of recurrence than PSA. This liquid biopsy approach, based on tumor-specific metabolic characteristics, may hold greater value compared to traditional methods. Studies have shown that in prostate cancer patients with biochemical recurrence, even when PSA levels are below 0.2 ng/mL, 68Ga-PSMA PET/CT can still detect lesions, suggesting that metabolically active lesions may exist before significant PSA elevation (58). This indirectly supports the concept of monitoring tumor metabolism. By capturing these early and specific metabolic signals, earlier intervention may be achieved, thereby improving patient prognosis.

### 5.3 Development of therapeutic strategies based on disulfidptosis

Therapeutic strategies targeting the mechanism of disulfidptosis are rapidly evolving, encompassing various forms such as small-molecule inhibitors and gene therapy. A long-established anti-alcohol dependence medication, disulfiram (DSF), is a potent aldehyde dehydrogenase (ALDH) inhibitor (60, 61). Inhibition of ALDH leads to the accumulation of toxic aldehydes, such as 4-hydroxynonenal, within cells, which can themselves cause oxidative damage (60). More importantly, disulfiram can directly deplete glutathione (GSH) and inhibit key enzymes including glucose-6-phosphate dehydrogenase (G6PD), thereby impairing the pentose phosphate pathway (62). By depleting GSH and suppressing NADPH production, it delivers a dual assault on the cellular antioxidant defense system. Upon NADPH depletion, cells lose their capacity to reduce oxidized glutathione (GSSG) to GSH, resulting in a sharp decline in the GSH/GSSG ratio and a surge in oxidative stress. Concurrently, they become unable to maintain the reduced state of the thiol (-SH) groups on cysteine residues of numerous functional proteins (54). In a highly oxidative environment, abnormal disulfide bond cross-linking occurs between these thiol groups or with other molecules, leading to loss of protein function, aggregation, and ultimately triggering disulfidptosis. Secondly, disulfiram has been repurposed for its antitumor activity observed across various cancers. Disulfiram is rapidly metabolized *in vivo* to diethyldithiocarbamate (DDC) (63). While DDC itself possesses biological activity, a significant enhancement of its antitumor efficacy relies on its chelation with divalent copper ions (Cu^2+^). Upon binding with Cu^2+^, DDC forms a highly lipophilic complex, Cu(DDC)_2_, also known as CuET, which is considered the primary active form responsible for disulfiram’s antitumor effects (64, 65). This complex readily traverses cell membranes to enter cells, enabling an *in situ* conversion from a “non-toxic to toxic” state within the tumor microenvironment for targeted chemotherapy (64, 66). Studies have confirmed that the combined use of disulfiram and copper (DSF/Cu) significantly enhances its cytotoxic effect against a variety of cancer cell types, with efficacy far superior to that of disulfiram alone (67–69). The molecular basis of this synergy lies precisely in the formation of the active Cu(DDC)_2_ complex, which interferes with critical intracellular metabolic pathways and signal transduction, ultimately leading to cell death. In renal clear cell carcinoma, disulfiram demonstrated potent antitumor activity by inhibiting NPL4, promoting oxidative stress, and inducing ferroptosis (70). Regarding combined immunotherapy, disulfiram has demonstrated the ability to enhance immune-related mechanisms. By activating immune-related pathways and increasing the immunogenicity of tumor cells, it holds promise for improving the efficacy of immunotherapies such as immune checkpoint inhibitors (71).

Furthermore, combination therapy with disulfiram and conventional chemotherapeutic agents (e.g., PARP inhibitors, sorafenib, doxorubicin) has demonstrated synergistic effects *in vitro* and in animal models, partially overcoming tumor resistance and improving therapeutic efficacy (70, 72, 73). The combined mechanism of action with PARP inhibitors primarily manifests in the enhancement of DNA damage and metabolic reprogramming. The disulfiram-copper chelate complex (CuET) formed upon the chelation of disulfiram with copper ions can trigger the integrated stress response, leading to early translational arrest and inducing nucleolar stress. These processes are accompanied by extensive DNA damage, such as single-strand breaks and base modifications (74), which create a superimposed effect with the PARP-DNA trapping induced by PARP inhibitors. PARP inhibitors, in turn, hinder the repair of single-strand breaks by inhibiting PARP enzyme activity, leading to replication fork stalling and ultimately the formation of lethal double-strand breaks (75). When the initial DNA damage caused by disulfiram is combined with the effect of PARP inhibitors blocking repair pathways, the genomic instability is drastically exacerbated, thereby exerting stronger cytotoxicity on tumor cells (76). Secondly, disulfiram can affect the self-renewal and drug resistance of cancer stem cells by inhibiting the activity of aldehyde dehydrogenase (ALDH) family member ALDH1A1. By eliminating tumor stem cell populations with high ALDH activity and combining with PARP inhibitors that target the bulk tumor cells, it can more thoroughly eradicate the tumor, prevent recurrence, and overcome PARP inhibitor resistance mediated by cancer stem cells (63).A similar mechanism is observed in its combination with doxorubicin. Doxorubicin induces DNA double-strand breaks by intercalating into the DNA double helix and interfering with topoisomerase II function (77). The CuET complex can trigger significant proteotoxic stress and genotoxic effects (74). This complex may cause additional damage on top of the DNA damage induced by doxorubicin by directly binding to DNA or interfering with the function of DNA repair-related proteins, forming more complex and difficult-to-repair DNA damage complexes. Unlike the previous two, the synergistic effect of disulfiram with sorafenib primarily manifests in enhancing anti-angiogenesis and inhibiting tumor invasion and metastasis. In terms of anti-angiogenesis, sorafenib directly targets and inhibits the function of vascular endothelial growth factor receptor (VEGFR) (78). CuET can effectively inhibit the activation of the nuclear factor-kappa B (NF-κB) signaling pathway (79), producing an additive effect with sorafenib’s mechanism of blocking VEGF signaling at the receptor level, thereby more effectively disrupting the tumor vascular network and inhibiting tumor blood supply and growth. Furthermore, CuET can inhibit the function of the ubiquitin-proteasome system (UPS) by targeting the p97-NPL4 axis (74). This action suppresses NF-κB activation, subsequently downregulating the expression of matrix metalloproteinases (MMPs) and epithelial-mesenchymal transition (EMT)-related markers (79). MMPs are key enzymes that degrade the extracellular matrix and promote tumor cell invasion and metastasis, while EMT is a crucial biological process by which tumor cells acquire migratory and invasive capabilities. Therefore, disulfiram effectively inhibits the invasive and metastatic potential of tumors through this mechanism, synergizing with the anti-metastatic effects inherent to sorafenib (78). The introduction of nanotechnology offers novel solutions for disulfiram drug delivery. The preliminary positive outcomes of disulfiram in cancer therapy have laid a foundation for its clinical translation, while simultaneously highlighting both its therapeutic potential and safety concerns in clinical application, underscoring the need for more in-depth consideration regarding the optimization of administration methods and dosage.

Through co-delivery via nanocarriers, precise release of disulfiram and metal ions can be achieved, enhancing drug stability and tumor targeting while reducing systemic toxicity (71, 80). This provides technical support for the widespread clinical application of disulfiram-based therapeutic strategies leveraging the disulfidptosis mechanism. In summary, therapeutic strategies based on disulfidptosis encompass small-molecule drugs and gene regulation. The synergistic effects of combining immunotherapy with conventional treatments also demonstrate considerable therapeutic potential. Nanodelivery systems provide new technical support for clinical translation, but further focus and development are needed in safety, dose optimization, and personalized treatment regimens (71, 80–82).

## 6 Conclusion and outlook

As a major threat to men’s health, urological cancers have shown an increasing incidence trend in recent years. Although disulfidptosis has demonstrated broad prospects as a novel cell death mechanism in cancer research, current studies still face numerous challenges. Insufficient in-depth analysis of the disulfidptosis mechanism has led many existing studies to focus on superficial phenomena, with inadequate systematic research on its regulatory networks, upstream/downstream signaling pathways, and specific intracellular targets (83). Furthermore, the lack of highly specific and sensitive *in vivo* detection methods limits its clinical application and efficacy monitoring. Disulfidptosis also necessitates more refined molecular models to elucidate its synergistic or antagonistic interactions with other death mechanisms (83). Furthermore, research on the interplay between disulfidptosis regulation and the immune system within heterogeneous tumor microenvironments remains limited. Future studies should explore these mechanisms through ongoing research and technological advancements, thereby providing more scientifically grounded guidance for both basic research and clinical applications.

This paper summarizes existing research findings on the clinical application and mechanisms of action of disulfidptosis in urological cancers. Disulfidptosis not only enriches the diversity of tumor cell death forms but also opens up entirely new approaches and strategies for the diagnosis and treatment of urological cancers. Therefore, the author reflects on its advantages and disadvantages: 1.Integrating nanotechnology with targeted drug delivery systems may be key to enhancing the safety and efficacy of disulfidptosis therapies. The precision release and targeted treatment enabled by such systems hold promise for overcoming issues like poor stability and significant side effects associated with traditional drugs. 2. Disulfidptosis regulates tumor cell survival and apoptosis through specific molecular pathways, revealing its complex role within the tumor microenvironment. These findings provide a theoretical foundation for developing drugs targeting apoptosis-related pathways, potentially enhancing treatment precision and efficacy. 3. While some studies highlight apoptosis’s potential in inhibiting tumor progression, caution is warranted regarding possible side effects and uncertainties arising from cellular environment changes. This underscores the need for future research to focus on detailed mechanism analysis, systematically evaluate its bidirectional regulatory effects, and avoid therapeutic risks stemming from a one-sided pursuit of single targets. In summary, the integration of multidisciplinary technologies, the construction of more comprehensive regulatory network models, the screening of advanced biomarkers, and the development of targeted drugs hold promise for making disulfidptosis a significant breakthrough in the treatment of urological cancers. It is hoped that a robust bridge between basic research and clinical application will transform disulfidptosis into a powerful tool for precision therapy in urological cancers, propelling this field to new heights.
